# Prospective Analysis of Hemorrhagic Cystitis and BK Viremia in Allogeneic Hematopoietic Stem Cell Transplantation

**DOI:** 10.4274/tjh.galenos.2019.2019.0296

**Published:** 2020-08-28

**Authors:** Erden Atilla, Can Ateş, Atilla Uslu, Pınar Ataca Atilla, Istar Dolapçı, Alper Tekeli, Pervin Topçuoğlu

**Affiliations:** 1 Ankara University Faculty of Medicine, Department of Hematology, Ankara, Turkey; 2 Van Yüzüncü Yıl University Faculty of Medicine, Department of Biostatistics, Van, Turkey; 3 Ankara University Faculty of Medicine, Department of Microbiology, Ankara, Turkey

**Keywords:** Hemorrhagic cystitis, BK viremia, Cytomegalovirus, Graft versus host disease

## Abstract

**Objective:**

BK virus (BKV) infection has been shown to be related to hemorrhagic cystitis (HC) in allogeneic hematopoietic stem cell transplantation (allo-HSCT). There are conflicting data regarding the association between BKV titers in plasma and clinical disease as well as the risk factors for BKV-related HC. Our aim is to study the risk factors and relationship with plasma BK viral load for development of HC in a prospective analysis.

**Materials and Methods:**

We prospectively evaluated 59 patients who received allo-HSCT between 2014 and 2016 by quantitative BK virus polymerase chain reaction (PCR) (Altona Diagnostics, Germany) from blood samples at days 0, 30, 60, and 90 after allo-HSCT. The patients were monitored for signs and symptoms of HC.

**Results:**

HC was diagnosed in 22 patients (37%) at a mean of 100 days (range: 0-367 days). In multivariate analysis, the usage of cyclophosphamide (sub-distribution hazard ratio [sdHR]: 7.82, confidence interval [CI]: 1.375-39.645, p=0.02), reactivated CMV (sdHR: 6.105, CI: 1.614-23.094, p=0.008), and positive BKV viremia (sdHR: 2.15, CI: 1.456-22.065, p=0.01) significantly increased the risk of developing HC. Patients with higher viral loads at day 30 and day 60 were diagnosed with more severe HC (p<0.001). Median BK viral loads of >101.5 copies/mL at day 0 (sensitivity 0.727, specificity 0.875), >98.5 copies/mL at day 30 (sensitivity 0.909, specificity 0.875), and >90.0 copies/mL at day 60 (sensitivity 0.909, specificity 0.875) were indicative of HC.

**Conclusion:**

Our study showed that administration of cyclophosphamide, CMV reactivation, and BK virus positivity were associated with HC. Plasma BK virus PCR titers at days 0, 30, and 60 after transplant were sensitive tools for predicting clinically proven HC.

## Abstract (tr)

**Amaç:**

BK virüs (BKV) enfeksiyonun allojeneik hematopoietik kök hücre naklinde (allo-HKHN) hemorajik sistit (HC) ile ilişkili olduğu gösterilmiştir. Literatürdeki çalışmalarda plazmadaki BKV titresi ile klinik hastalık arasında ve BKV ilişkili HC risk faktörleri açısından çelişkili veriler mevcuttur. Amacımız, prospektif olarak HC gelişimi risk faktörleri ile plazma BK viral yükü arasındaki ilişkinin gösterilmesidir.

**Gereç ve Yöntemler:**

2014-2016 yılları arasında 59 allo-HKHN alıcısının 0, 30, 60 ve 90. günlerdeki kan örneklerinden kantitatif olarak BK virüs polimeraz zincir reaksiyonu (PCR) (Altona Diagnostics, Almanya) değerlendirilmiştir. Hastalar HC belirtileri ve semptomaları açısından izlenmiştir.

**Bulgular:**

Yirmi iki hastada (%37) nakil sonrası ortalama 100. günde (0-367 gün) HC tanısı konmuştur. Çok değişkenli analizde (siklosfosfamid alan [SdHR]: 7,82, güven aralığı [GA]: 1,375-39,645, p=0,02), CMV reaktivasyonu geçiren (SdHR: 6,105, GA: 1,614-23,094, p=0,008) ve BK virüs pozitifliği (SdHR: 2,15, GA: 1,456-22,065, p=0,01) olan hastalarda artmış HC riski saptanmıştır. Viral yükleri 30. ve 60. günlerde yüksek tespit edilen hastalarda daha ciddi HC görülmüştür (p<0,001). Ortanca BK virüs yükü 0. günde >101,5 kopya/mL (sensitivite 0,727, spesifite 0,875), 30. günde > 98,5 kopya/mL (sensitivite 0,909, spesifite 0,875), 90. günde >90,0 kopya/mL (sensitivite 0,909, spesifite 0,875) ise bu durumlar HC göstergesi olabilir.

**Sonuç:**

Çalışmamızda siklofosfamid, CMV reaktivasyonu ve BK virüs pozitifliği HC ile ilişkili olduğu gösterilmiştir. Plazma BK virüs PCR titleri transplant sonrası 0, 30, 60. günlerde klinik olarak kanıtlanmış HC için hassas araçlardır.

## Introduction

Hemorrhagic cystitis (HC) is a cause of morbidity and mortality that occurs in 10%-25% of hematopoietic stem cell transplantation (HSCT) recipients [1,2]; some studies have reported an incidence of up to 70% [3,4]. Symptoms vary from microscopic hematuria to severe obstructive nephropathy [5]. Early hematuria is usually the result of chemotherapy toxicity; however, late-occurring HC is multifactorial. The risk factors for HC include the type of conditioning, timing of engraftment, usage of cyclophosphamide (Cy), development of graft versus host disease (GVHD), presence of BK virus (BKV) infection and other viral infections, advanced age at transplantation, and thrombocytopenia or coagulopathy [6,7,8,9,10,11].

*Polyomavirus hominis* 1, also called BK virus, is a non-enveloped, encapsulated DNA virus in the family *Papovaviridae *[1]. The shedding of latent BKV is frequently detected in immunocompromised individuals [12,13]. The association of BKV-related HC in HSCT settings was first reported by Arthur et al. [14,15]. Asymptomatic BKV shedding without clinical relevance might be detected by polymerase chain reaction (PCR) in urine [16,17]; BK infection generally develops in more than 50% of allo-HSCT patients in peri-engraftment weeks, but overt HC occurs in about 20% of patients [17]. There is a conflicting relationship between BKV titers in plasma and clinical disease, which may come from discrepancies between studies [18,19]. A high-level of BK viremia (>3-4 log_10_ copies/mL) has been shown to be correlated to BKV-HC [20]. Our aims in this prospective study are to detect the risk factors for HC following allo-HSCT and to illustrate the relationship between plasma BK viral load and HC.

## Materials and Methods

We included 59 adult patients who underwent allo-HSCT between 2014 and 2016 for any hematological disease at our institute. Institutional ethical board approval and the informed consent of all participants were obtained. This project was supported by grant number 15B0230007.

Patients were monitored for signs and symptoms of HC at initial admission for allo-HSCT and routinely in outpatient visits. Late-onset HC was generally defined as HC occurring more than one week after transplant [21]. Hematuria was defined as >5 red blood cells in high-powered field microscopy, or documented gross hematuria with or without symptoms of cystitis. The grade of hematuria was defined according to criteria described by Bedi et al. [2]. A diagnosis of HC was made when clinically significant macroscopic hematuria (grade 2 or higher) was present. All patients received standard supportive care, including antifungal, antiviral, and antibacterial therapy (ciprofloxacin) for prophylaxis prior to allo-HSCT.

Patient plasma samples were collected prospectively prior to allo-HSCT and on days 30, 60, and 90 after transplant, as well as in each HC attack, and stored at -96 °C. BK virus testing was performed in the microbiology laboratory. Viral DNA was extracted with a QIAmp DNA Mini Kit, and viral load was detected with a Real Star BKV and JCV PCR Kit (Altona Diagnostics, Germany) with the real-time polymerase chain reaction method in a Rotor-Gene Q/6000 (QIAGEN, Germany). The concentration of BKV DNA molecules that can be assigned with a positivity rate of ≥95% and determined by probit analysis is 0.712 copies/µL. BKV PCR was considered positive if any viral copies were identified. Patients were diagnosed with BKV-associated HC when they had grade 2 or higher hematuria in addition to positive BKV PCR.

To compare patients with or without occurrence of HC, chi-square and Fisher exact tests were used where appropriate. Risk factors for the development of BKV-HC were evaluated first in univariate and then in multivariate analyses. The Spearman correlation (rho) test was used to evaluate the relationship between HC grades and BK viral loads. ROC analysis was performed to determine the positive thresholds of BK viral loads. The most specific and sensitive points under the line were detected by the Youden index. A p-value of less than 0.05 was considered statistically significant. The statistical analyses were performed using SPSS 20.0 (IBM Corp., Armonk, NY, USA).

## Results

The median age of the participants was 41 (range: 22-71) years; 18 patients (31%) were aged >50. The male/female ratio was 1.36 (34/25). Malignant hematological disease (acute myeloid leukemia [AML], acute lymphoblastic leukemia [ALL], or myelodysplastic syndrome [MDS]) was diagnosed in 52 patients (88%). The stem cell source was peripheral blood in 52 patients (88%) and bone marrow in 7 patients (12%). The stem cells came from HLA-matched related donors for 23 patients (39%), haploidentical donors for 5 patients (8%), and unrelated donors for 31 patients (53%). A myeloablative conditioning regimen was administered for 37 patients (63%), and 44 patients (75%) received cyclophosphamide (Cy). The most common GVHD prophylaxis administered was cyclosporine (CSA) plus methotrexate (Mtx) in 49 patients (83%), followed by mycophenolate mofetil (MMF) plus Mtx in 5 patients (8%). Five patients (8%) underwent haploidentical allo-HSCT and received tacrolimus, MMF, and Cy for GVHD prophylaxis. Acute GVHD was diagnosed in 38 patients (64%) at a median time of 67 days: grade I-II gastrointestinal/skin/liver in 31 patients (84%) and grade III-IV gastrointestinal/skin/liver in 6 patients (16%). The median times to neutrophil and platelet engraftment were 20 days (range: 11-45) and 21 days (range: 9-48), respectively. Patient characteristics are summarized in Table 1.

HC was documented in 22 patients (37%) at a median of 100 days after allo-HSCT (range: 0-367 days). The distribution of HC grades was as follows: 13/22 (59%) grade 2, 2/22 (9%) grade 3, and 7/22 (32%) grade 4. Late-onset HC occurred in 18 patients (31%). The median platelet count at the onset of HC was 52x10^9^/L (range: 8-372x10^9^/L). Ten patients (17%) developed HC before platelet and neutrophil engraftment. None of the patients had pro-hemorrhagic abnormality. Positive CMV reactivation was detected coincidently during HC episodes in 18 patients (31%) at a median time of 72 days. In univariate analysis, myeloablative conditioning regimen, Cy administration, presence of acute GVHD, CMV reactivation, and BK viremia were associated with a higher risk of HC (p=0.022, p=0.026, p=0.031, p=0.012, and p<0.0001 respectively). In multivariate analysis, patients that received Cy (sub-distribution hazard ratio (sdHR): 7.82, CI: 1.375-39.645, p=0.020) had CMV reactivation (sdHR: 6.105, CI: 1.614-23.094, p=0.008), and were positive for BKV viremia (sdHR: 2.15, CI: 1.456-22.065, p=0.01) had significantly increased risk of developing HC (Table 2). HC occurred in 3 of 5 haploidentical allo-HSCT patients who received Cy at both conditioning and GVHD prophylaxis. Therefore, more detailed analysis of excess Cy was not possible.

BKV DNA assay positivity (copies/mL >0) in plasma was shown in at least one measurement in 30 patients (51%). Eighteen of 59 patients (31%) developed BKV-related HC during follow-up. The frequency of plasma BKV positivity and the median number of viral copies/mL at days 0, 30, 60, and 90 are given in detail in Table 3. The numbers of patients that had HC at days 0, 30, 60, and 90 were 4, 16, 14, and 7. The frequency of BKV positivity was increased in patients with late onset HC (day 0: 4/15 (26%), day 30: 16/24 (66%), day 60: 14/16 (87%), day 90: 7/7 (100%); p=0.007).

We also investigated the predictive value of the BK viral load at day 0, 30, 60, and 90 for the severity of hematuria. Patients with higher viral loads at days 30 and 60 were diagnosed with more severe hematuria (p<0.001). The correlation between viral load and grade of hematuria grew stronger from day 0 to day 60 and then weaker from day 60 to day 90 (Table 4). According to ROC curve analysis, we suggest that median BK viral load of >101.5 copies/mL at day 0 (sensitivity 0.727, specificity 0.875), median BK viral load of >98.5 copies/mL at day 30 (sensitivity 0.909, specificity 0.875), and median BK viral load of >90.0 copies/mL at day 60 (sensitivity 0.909, specificity 0.875) are indicative of HC (Table 5).

Upon the occurrence of HC, intravenous hydration was initiated for each patient. Nine of 22 patients (HC of ≥grade 3) had continuous bladder irrigation through a bladder catheter. Platelet and RBC transfusions were administered to maintain platelets at >50x10^9^/L and hematocrit at >25. HC was resolved in 14 patients (64%). Eight BKV-related HC patients received 0.5-1 mg/kg intravenous cidofovir once every two weeks due to persistence of hematuria (7 patients had grade 4 hematuria and 1 patient had grade 3 hematuria) and increasing viral load. The median time to cidofovir treatment since the beginning of HC was 2 months (range: 1-3). Six of these 8 patients responded after a median of 2 doses (range: 1-5) of cidofovir treatment (75%). Probenecid could not be administered because it was not available. None of the patients had renal compromise. Two unresponsive patients with grade 4 hematuria underwent mucous electrocoagulation and macroscopic hematuria disappeared. The median time from allo-HSCT to last follow-up was 14 months (range: 1-34). No statistically significant difference was detected in terms of survival in patients with or without HC.

## Discussion

This prospectively designed study evaluates the risk factors for HC and illustrates the relationship between BK viremia and HC in allo-HSCT recipients. HC is a considerable cause of morbidity associated with prolonged hospitalization and urinary obstruction in severe cases after allo-HSCT [22]. HC was detected in 22 patients (37%) at a median of 100 days after allo-HSCT (minimum-maximum: 0-367 days) in our study. The incidence of early HC in patients treated with normal doses of Cy who were adequately hydrated is 10% [23]. Despite the ability to decrease incidence through sufficient hydration and the use of mesna [24], conditioning-induced early-onset HC is still a problem and occurred in 7% of our patients. We found increased risk for HC with myeloablative conditioning and the presence of acute GVHD in univariate analysis, and the use of Cy and the occurrence of CMV and BKV in multivariate analysis, similar to prior reports [16,24,25,26].

The BK virus has been postulated to reactivate from a latent state in immunocompromised patients in the etiology of HC in allo-HCST recipients. The proposed pathogenesis of BKV-related HC occurs in three steps: 1) chemotherapy/irradiation damages the uroepithelium and decreases BKV-specific cellular immunity; 2) BKV replicates during the immunosuppressive phase; and 3) the immune attack further damages the uroepithelium after hematopoietic reconstitution [9]. To clarify the timing and severity of BKV-related HC, plasma BK viral loads at days 0, 30, 60, and 90 were included in our study. In agreement with the three-phase theory, we detected clinical BKV-related HC more frequently following engraftment. The correlation between the severity of HC and the plasma BK viral load was statistically significant at days 30 and 60. Some patients in our study had early-onset BKV-related HC, but it is essential to consider that the patients involved in the study were diagnosed mostly with malignant disease and received chemotherapy and immunosuppression prior to allo-HSCT. On the other hand, BKV DNA was also identified in healthy donor leukocytes and bone marrow cells [27]. In order to test the possibility of virus transmission during blood product transfusion or allo-HSCT, the BKV genotypes from both donors and patients were evaluated. Leung et al. demonstrated that patients with severe HC shared the donor BKV genotype, suggesting that transmission might be involved during transplantation [28]. Transmission or prior immunosuppression may explain our patients having early presentation of BKV-related HC prior to engraftment.

The incidence of BK viruria is about 50% following allo-HSCT [5]. Patients with HC are prone to higher peak urine BK viral loads [11,19,29]. BK viruria of >10^7 ^copies/mL is one of the diagnostic criteria for BK-related HC with a sensitivity of 86% and a specificity of 60% [20]. At the same time, in 40%-50% of HSCT patients with persistent viruria, HC did not develop [2,29]. Asymptomatic viral shedding in urine is found even in 5% of healthy individuals [30]. The positive predictive value of viremia is better than that of viruria for the occurrence of HC [1,31]. The ECIL guidelines state that plasma BK virus loads of >3-4log_10_ copies/mL play a role in the management and follow-up of allo-HSCT recipients [20]. Viremia of >10^4^ copies/mL for more than 3 weeks particularly contributes to HC in kidney transplant recipients [32]. Erard et al. [17] showed in a multivariate model that plasma viral load of more than 10^4^ copies/mL was associated with developing HC in allogeneic hematopoietic stem cell transplant recipients. Höller et al.  [33] could not detect a predictive cutoff for plasma viral load in a recent study, but they indicated that the presence of viremia might itself predict BKV-associated morbidity. Interestingly, we detected a lower threshold (2 log_10_) for BK-related HC in our prospective study, which might be related to the limited number of patients included in our study. We believe that closer follow-up should be considered for high-risk patients.

BK virus infection immune control depends on both CD4+ and CD8+ T-cells [34]. Although previous reports failed to demonstrate a correlation between GVHD and HC [2,30,35], we found a positive correlation in a univariate model. The immunosuppression of GVHD and immunosuppressive treatment both contribute to mucosal damage and may cause uncontrolled proliferation of the BK virus. Similarly, CMV viremia causes impaired T-cell immunity and weak T-cell responses [36]. Interestingly, heterologous transcriptional transactivation of CMV by the BKV antigen was proposed to connect these two agents [37]. It is possible that the presence of GVHD or CMV viremia may contribute to BK virus-related HC at lower BKV thresholds in our study. Since BK virus infections are activations of latent viral infections rather than primary infections, BK virus IgG titers are a possible marker of latent infection [38].

The treatment of BKV-associated HC is still controversial. Cidofovir has been demonstrated to be active against BKV in vitro and in clinical studies [39,40,41,42]. Savona et al. [42] showed that 84% of patients clinically responded to cidofovir; however, only 47% had decreased viral load in the urine. The timing of application varies; the most widely accepted is to consider cidofovir in persistent hematuria for 2 weeks with a significant increase in viral load [6]. Eight patients received cidofovir in our study, in agreement with the literature. Leflunomide is an anti-CMV agent and may be effective in BK virus-infected patients [43]. Indeed, fluoroquinolones may be preferred as prophylactic agents against the BK virus with modest activity and a low selectivity index [44]. Tang et al. [45] demonstrated that surgical treatments, including embolization and mucous electrocoagulation, are safe and effective in severe refractory HC. Recently, off-the-shelf virus-specific T cells were demonstrated to be a safe and effective broad-spectrum approach in severe viral infections after allo-HSCT [46]. BKV is a common cause of HC in HSCT, and it is associated with increased hospital costs due to prolonged hospitalization, a 2- to 3-fold increase in RBC and platelet transfusions, and cidofovir treatment if needed [47].

## Conclusion

HC is a common complication of allo-HSCT that remains a challenge. Our prospective data confirmed that administration of Cy, myeloablative conditioning, the presence of GVHD, CMV reactivation, and BKV infection are risk factors for HC in allo-HSCT recipients. The sensitivity and specificity of the plasma BK PCR test was increased in later periods after transplant for diagnosis of BKV-related HC. Routine plasma BK viral monitoring at days 0, 30, and 60 after transplant may assist in the diagnosis and treatment of BKV-related HC.

## Figures and Tables

**Table 1:**
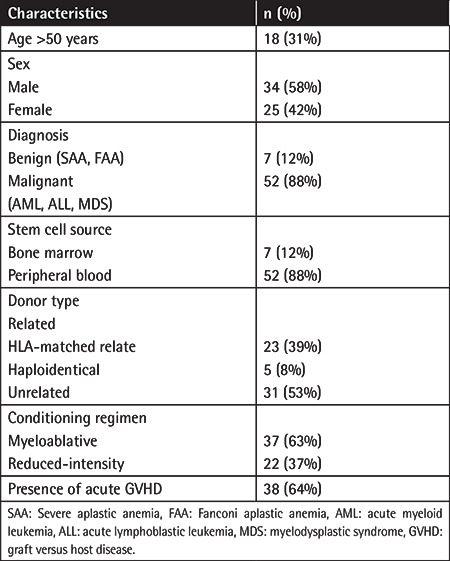
Patient characteristics.

**Table 2:**
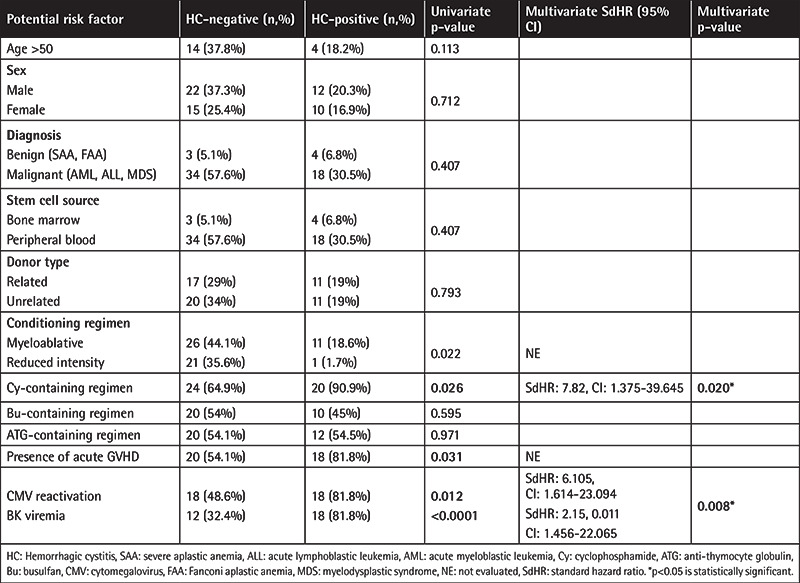
Univariate and multivariate analysis of risk factors associated with HC.

**Table 3:**
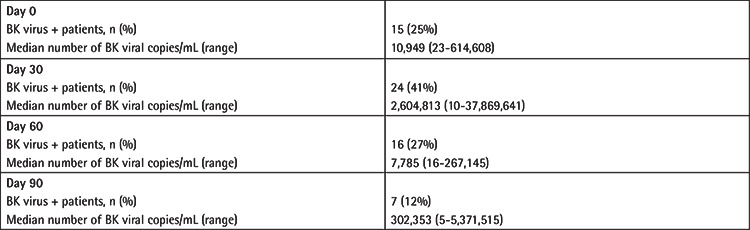
Frequency of BK viremia patients (n=59) and median number of BK viral copies/mL at days 0, 30, 60, and 90.

**Table 4:**
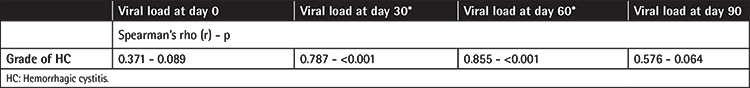
Correlation between grade of hematuria and BK viral load (copies/mL) (Spearman’s rho). The correlation between viral load and grade of hematuria increased from day 0 to day 60 and then weakened between day 60 and day 90.

**Table 5:**
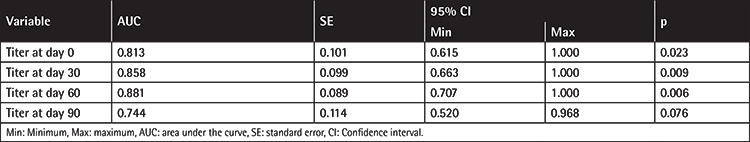
The diagnostic power of hemorrhagic cystitis (HC) for 4 different titers as evaluated by ROC analysis. According to the area under the curve, the best diagnostic capabilities for HC were at days 60, 30, and 0, respectively.
